# Direct and indirect costs of Multiple Sclerosis in Baix Llobregat (Catalonia, Spain), according to disability

**DOI:** 10.1186/1472-6963-6-143

**Published:** 2006-11-01

**Authors:** Virginia Casado, Sergio Martínez-Yélamos, Antonio Martínez-Yélamos, Olga Carmona, Lucia Alonso, Lucia Romero, Esther Moral, Laura Gubieras, Txomin Arbizu

**Affiliations:** 1 Multiple Sclerosis Unit, Institute of Biomedical Investigation, University Hospital of Bellvitge, Faculty of Medicine, Campus Bellvitge, University of Barcelona (IDIBELL), Barcelona, Spain; 2 Neurology Department, Hospital de San Lorenzo, Viladecans, Barcelona, Spain; 3 Neurology Department, Hospital de Figueres, Girona, Spain

## Abstract

**Background:**

Multiple sclerosis (MS) is an incurable chronic disease that predominantly affects young adults. It has a high socio-economic impact which increases as disability progresses. An assessment of the real costs of MS may contribute to our knowledge of the disease and to treat it more efficiently. Our objective is to assess the direct and indirect costs of MS from a societal perspective, in patients monitored in our MS Unit (Baix Llobregat, Catalonia) and grouped according to their disability (EDSS).

**Methods:**

We analysed data from 200 MS patients, who answered a questionnaire on resource consumption, employment and economical status. Mean age was 41.6 years, mean EDSS 2.7, 65.5% of patients were female, 79.5% had a relapsing-remitting course, and 67.5% of them were receiving immunomodulatory treatment (IT). Patients were grouped into five EDSS stages. Data from the questionnaires, hospital charts, Catalan Health Service tariffs, and figures from Catalan Institute of Statistics were used to calculate the direct and indirect costs. The cost-of-illness method, and the human capital approach for indirect costs, were applied. Sensitivity analyses were performed to strengthen results.

**Results:**

The mean total annual cost of MS per patient results 24272 euros. This cost varied according to EDSS: 14327 euros (EDSS = 0), 18837 euros (EDSS = 1–3), 27870 euros (EDSS = 3.5–5.5), 41198 euros (EDSS = 6–7) and 52841 euros (EDSS>7.5). When the mean total annual costs was adjusted by the mean % of patients on IT in our Unit (31%) the result was 19589 euros. The key-drivers for direct costs were IT in low EDSS stages, and caregiver costs in high stages. Indirect costs were assessed in terms of the loss of productivity when patients stop working. Direct costs accounted for around 60% of total costs in all EDSS groups. IT accounts from 78% to 11% of direct costs, and decreased as disability progressed.

**Conclusion:**

The total mean social costs of MS in a cohort from Baix Llobregat (Catalonia) were estimated at 24272 euros per patient/year, and ranged between 14327 euros (EDSS = 0) and 52841 euros (EDSS = 7.5–9.5). Total costs, and particularly informal and direct costs, increased as the disability progressed. IT should be able to delay the progression of disability to be efficient and not only effective.

## Background

Multiple sclerosis (MS) is a chronic, demyelinating inflammatory disease of the central nervous system (CNS). Its aetiology is still unknown. In Spain, the prevalence of MS is 53–58 per 100000 [1,2]. It predominantly affects young adults, with onset in third decade of life, and females (ratio 2:1).

The prognosis for MS is unclear and is probably influenced by genetic, biochemical and clinical factors. Life expectancy seems to be relatively unaffected by MS, but morbidity is considerable: 50% of patients will need help to walk 10 years after diagnosis, according to natural history studies[3]. However, recent studies show that the disease has a more benign evolution[4].

MS represents the second most common cause of disability in young adults, after road accidents, and has a high socioeconomic impact.

In the last decade immunomodulatory treatments (IT) have demonstrated their efficacy [5-8] in reducing the number of relapses and, to a lesser extent, in slowing the progression of the disability. However, these tendencies have still not been confirmed for the long term. As treatments are expensive, a question arises: are they efficient, as well as being effective? In other words, because of their beneficial effect and despite their elevated costs, is IT more cost-saving than other therapeutic options in the long term? Many cost-effectiveness analyses have been published [9-17] with controversial results.

It is essential to assess the real cost of MS if we are to make a cost-effectiveness analysis whose results can be applied to daily clinical practice. Cost-utility analysis, a particular type of cost-effectiveness analysis in which outcomes are measured in terms of quality of life, may be particularly appropriate for a disease like MS, as the assessment of quality of life integrates physical and psychological components of wellbeing which are affected by the disease [18]. The cost of MS has been calculated in several countries [19-32]. These studies have shown the significant economic impact of the disease and how it increases as disability progresses. Cost-of-illness studies are of particular interest because they show costs of every item related to the disease, thus revealing the main factors that contribute to the total costs.

Our study, the first of this type in our region (Catalonia, Spain), attempts to assess the overall cost to society of MS, calculated from a population of patients monitored in our MS Unit. The approach that we used could be extended to assess MS costs nationwide. Our intention was also to show how the costs of MS change as the disease progresses. Therefore, costs were calculated in different stages of disability as measured by the EDSS (*Expanded Disability Status Scale*).

A prevalence cost-of-illness analysis, like the one we present, measures a disease's baseline costs to society. Such an analysis could be useful for making decisions on research investments. In contrast, incidence based cost studies are more useful when estimating the effect of a treatment on future costs.

## Methods

We performed an observational cost-of-illness study [33,34] for MS, a chronic disease, using a prevalence approach, which entails estimating all costs for a particular population. We calculated the cost of MS per patient over a given period of time (1 year) in a specific geographical area (Baix Llobregat, Catalonia, Spain). We estimated costs for a group of patients whose results could be extrapolated to the general population (a "bottom-up" approach). Data were collected in those patients retrospectively, as they referred to what occurred previous to the moment when they answered the questionnaire elaborated. A "top down" approach would have involved using data from database and statistical registers. Such an approach is not suitable for a chronic disease like MS. It would tend to underestimate total costs, as information would not be available for calculating every cost in the patient population (e.g. indirect costs).

### Patients'

We calculated direct and indirect costs due to MS for a group of 200 patients monitored in our MS Unit, who answered, consecutively, a questionnaire elaborated for that purpose. Recently diagnosed patients were excluded from the study, as a complex, disability-focused questionnaire could have given them a negative perception of their disease. Patients who were participating in clinical trials at the time of the study were also excluded, as they tend to consume more resources than the general MS population (outpatients' visits, tests...). We considered the clinical and demographic characteristics of the patients in the study to be similar to those of the general MS population seen in our clinics. No statistically significant differences were found between patients who answered the questionnaire and patients who did not (except for the percentage of patients on IT, see Table 1).

Patients' levels of disability were measured with the EDSS. Patients were then put into five groups, representing clinically relevant different disease stages: stage I (EDSS = 0, the patient is not disabled but has been diagnosed of a chronic disease): 23 patients; stage II (EDSS = 1–3, minimally disabled): 107 patients; stage III (EDSS 3.5–5.5, rather disabled): 42 patients; stage IV (EDSS = 6–7, patient still capable of walking with aid): 17 patients; and stage V (EDSS = 7.5–9.5, patient is unable to walk at all): 11 patients. Patients' EDSS scores were obtained from their medical charts.

### Data collection

The questionnaire was anonymous and voluntary. It was mailed to 454 patients with a statement of informed consent to be signed by patients and caregivers. A letter explaining the purpose of the study was also sent to every patient. We obtained 200 responses (44%). Data were collected on socio-demographic and clinical characteristics; work-status; resource consumption; and costs attributable to MS during the previous 15–365 days. The length of the recall period varied according to the item in question, to minimize any possible bias due to a patient's lack of memory. The recall periods for the different items were as follows:

-previous year for hospitalisation, need for caregivers and sick leave, due to MS

-previous 3 months for outpatient visits

-previous 15 days for drug treatments, rehabilitation, visits to the General Practitioner, ambulance displacements, laboratory-imaging tests, and days of leisure time and/or daily activities lost due to MS.

A recall period of 1–3 months has been shown to be generally reliable for retrospective data. For more important or less frequent events, such as hospitalisation, a 12-month recall period is considered appropriate [25-27,30,32].

Questions about home modifications referred to the full duration of the disease. The unit costs for adaptations were not available for our region. Therefore, we used the mean cost per patient per year of home adaptations made because of MS, which has previously been calculated in the European Community [25-27,29].

Data obtained from questionnaires were verified and completed with data taken from inpatient records, medical files and the Medical Records Department, to improve reliability of the patients' responses. Data were then annualised when necessary. The study period was one year, from 1-8-2001 to 31-7-2002.

The Ethical Committee of the University Hospital of Bellvitge and the Institute for Biomedical Investigation of Bellvitge (Barcelona, Spain), where the study was carried out, gave their approval for the whole project.

### Unit costs

Unit costs were obtained from: Catalan Health Institute tariffs (costs of: procedures, visits to physicians, hospitalisations); the Catalan Institute of Statistics (the minimum hourly wage, the average costs of labour in each sector of activity, the median time to find a job in Spain); market prices for pharmacological treatments (*Drugs and Chemists Pharmacy Database of the General Council of Official Colleges of Pharmacists, 2004*); and investments required to overcome architectural barriers. Every unit cost was updated to the equivalent number of euros in 2004, as the resources consumed in 2001 and 2004 did not differ greatly.

### Direct costs

We calculated the direct costs by multiplying the resources consumed per patient by their unit costs. The costs of hospitalisation, ambulatory assistance, treatments, rehabilitation, transport, investments to overcome architectural barriers, disability aids, and informal care (provided by a non-paid caregiver who was a relative in most cases) are included as direct costs [25-27,30].

### Indirect costs

We used a Human Capital Approach to estimate the indirect costs. This approach involves analysing MS-attributable loss of productivity, in terms of morbidity and mortality. The number of days lost due to sick leave and changes in the work status of patients and caregivers because of MS are considered to be morbidity. Mortality, in terms of life expectancy, is not significantly affected by MS. Therefore it was considered irrelevant (equivalent to 0) in this study. A minimum hourly wage was applied to patients who did not carry out "productive activities" in the labour market (*e.g*, housewives), to avoid underestimating indirect costs in this group of patients. For the rest of the patients, we applied the salary cost per hour in each professional sector, as described in the "*National list of Economic Activities in fourth trimester 2003 – CNAE 2003*". Thus, the results obtained should be considered a minimum estimation of indirect costs.

### Informal care costs

Informal care is provided by non-paid caregivers, who are generally patients' family members. The costs of this informal care were included in the direct costs [25-27,30]. We used a replacement cost of 50% of the minimum hourly wage [19,30,32] This figure was selected as empirical studies have estimated the value of unpaid labour as being between 0.4 and 0.6 of potential earned income [19]. The replacement cost was then multiplied by the number of hours employed by the caregiver in taking care of the patient. A specific questionnaire was answered by the patient's caregiver, who reported the number of hours employed in this activity. As the replacement cost is not clearly standardized, a sensitivity analysis was performed. Results are shown in Table 2. This analysis involved valuing caregiver time at 30% and 100% of the minimum hourly wage (extreme values previously employed in literature, [7,24,25]).

### Ethical approval

The project obtained the approval of the Ethics Committees for the Hospital Universitario de Bellvitge and the Instituto de Investigación Biomédica de Bellvitge (IDIBELL), Barcelona, Spain.

All statistical analysis were performed using SPSS software. X-^*2*^, Student's *t *and U- Mann-Whitney tests were used.

## Results

We analysed data from 200 patients with definite MS. The average age was 41.6 (SD: 10.7), 65.5% of patients were female, and 78.5% had relapsing-remitting MS. The mean EDSS was 2.7 (SD: 2.2) and the median EDSS was 2. The mean number of years from MS onset was 12.3 (SD: 8.39) (Table 1).

All costs were calculated per patient per year in each EDSS group. The mean cost per patient was calculated from the total cost divided by the number of patients included in the study.

Direct costs (Table 2) ranged from 8706 and 37062 euros per patient per year, depending on the EDSS. Direct costs were mostly due to pharmacological treatment (67.5% of patients were on IT in the sample studied) and to informal care. IT represented up to 78% of all direct costs in the lowest EDSS stages. However, it only represented 11% of all direct costs when patients reached EDSS >7. At this stage, rehabilitation, investments to overcome architectural barriers, disability aids and particularly informal care contributed most to total direct costs (informal care costs represent 60.4% of direct costs when EDSS >7). After a sensitivity analysis, the overall trend remained as in the primary analysis (Table 2): direct costs increased as disability progressed, and were higher than indirect costs.

Indirect costs (Table 3) reflect the productivity lost by patients, caused by "short term sickness absence" (days of sick leave) and "long term sickness-absence" (invalidity, unemployment and other changes in work status caused by MS). Indirect costs ranged from 5621 to 17161 euros per patient per year. They increased as the EDSS stage increased, and represented 34.6% of total costs. The key-drivers of these costs were MS-attributable changes in patients' work-status.

The total costs of MS per patient per year, including the direct and indirect costs, are summarised in Table 4. The mean annual total direct and indirect costs for an MS patient in the sample studied (67.5% of patients on IT) was 24272 euros. When we adjusted this figure to the percentage of patients receiving IT in our MS Unit (31%), the total was 19589 euros. IT accounted for 34.5% of total costs in the sample studied, and for 19% in our MS Unit (mean per patient per year).

## Discussion

The aim of this study was to perform a complete analysis of the costs of MS, and to describe how costs change in different disability stages.

We have chosen a prevalence approach that is "bottom-up", as described in the method section. We performed the study from a social perspective, as this meant that all costs generated by MS could be included (health-care and non-health-care costs) independently of who pays for them. This is the most extensive approach, and enables data to be disaggregated for further analyses. This approach also enabled us to classify costs according to disability. In addition, we could obtain the costs derived from each disease-related item separately, which may be useful when establishing adequate therapeutic strategies and making cost-effectiveness analyses. An important disadvantage of this approach is the difficulty involved in selecting a representative sample. However, the alternative "top-down" approaches (when data about diagnoses and resources consumed because of MS are obtained from published sources), also have a significant disadvantage: available data are more limited and mostly retrospective. The sample we analysed in this study is representative of the MS population attended in our MS Unit. It included patients from the entire spectrum of the disease (66 % with very mild MS, EDSS<3.5; 4% wheelchair bound; 1.5% bed-ridden; 7.5% with the primary progressive form).

The main purpose of the study was to obtain the costs of MS at different levels of disability. Therefore, we decided to group patients into clinically relevant stages of disability, in order to see whether the costs varied significantly among such stages (even though some groups contained a small number of patients). There were marked differences in costs among the five disability stages, particularly for direct costs (Table 2). The differences among the stages were less marked for indirect costs (Table 3). Total costs increased as the EDSS stage increased (Tables 2, 3, 4). Indirect costs were a little higher in stage IV than V (more disabled patients). This is probably due to the small number of patients in these groups. However, it also reflects the fact that indirect costs may not differ much between patients with EDSS ≥6 or ≥7.5. In both of these stages, most patients are unable to work and their productivity losses are therefore similar. It is interesting to note that even when patients have no disability (EDSS = 0), the direct and indirect costs caused by MS are 14327 euros per patient/year. Another surprising finding is that disabled patients, with EDSS 3.5–5.5, did not use ambulances. This is probably because such patients used taxis for transport, as described in Table 2. The small sample size may have contributed to this result.

The methodology used in our study has also been widely employed in the literature. Therefore, comparisons of our results with those obtained from similar studies carried out in other countries may be possible. However, direct comparisons with available data from other costs studies can not be made because of differences in healthcare systems, healthcare usage, absolute and relative costs.

Major limitations of the study include: the retrospective collection of data, and the small number of patients involved (200). There was a low response rate to the questionnaire, which may have been due to the questionnaire's complexity. We did not carry out a second mailing, and did not personally insist on patients responding in order to guarantee their willingness. The fact that we did not find statistically significant differences between patients who responded to the questionnaire and those who did not minimizes the possibility of a selection bias.

There was a higher proportion of patients on IT (67.5%) in our sample than in the general MS population. This was probably because treated patients tend to visit our MS Unit frequently (for medical visits, collection of medication, procedures etc.). In addition, they may feel more involved with the MS Unit and therefore may be more willing to answer the questionnaire (47.5% of patients who did not answer the questionnaire were on IT). We made a rough estimation of costs for the general MS population by adjusting the costs [32] of IT to the percentage of IT-treated patients in our MS Unit (specialised Unit at a University Hospital). The results are detailed in Table 4. They show that costs increased as EDSS stage increased, as in the primary analysis.

Another limitation of the study was the lack of information on costs attributable to MS-associated neuropsychiatric complications and cognitive impairment. Such costs were not specifically measured in our patients, even though cognitive and neuropsychiatric factors have been shown to have a significant impact on MS patients [18].

There are many controversial aspects in the methodology employed. Firstly, the "Human Capital Approach" has a significant limitation. It tends to underestimate the costs for people who are not in paid employment at the time of the study. Therefore, results obtained using this approach have to be considered a minimum estimation of costs. In spite of this, it is the most commonly used method in the literature. Secondly, if tariffs are used as unit costs when no other opportunity costs are available -as in our case- costs can be overestimated (by approximately 20%, as reported in United Kingdom)[27] or underestimated (as in Germany, where tariffs appear to include incentives for a reduction in usage and are therefore lower than opportunity costs)[26]. This issue has not been resolved in Spain, and may therefore lead to either an overestimation or underestimation of total costs for society. Finally, informal care costs can be evaluated in different ways. In our case, only a minority of caregivers (0–36%, depending on EDSS stage) had to give up their remunerated work because of the patient's MS, thus leading to productivity losses (indirect costs). Therefore, in order to be consistent and to obtain a better reflection of the informal care costs in our sample, we considered informal care as a direct cost. We used the replacement method to measure this cost. A sensitivity analysis was performed by varying the replacement costs used, which strengthened the results obtained in the primary analysis.

We consider that this study represents an exhaustive estimation of the real costs of MS. It shows that the direct costs are higher than indirect costs, and that the overall costs increase as the disability progresses. The main factors that contribute to direct costs differ depending on the EDSS stage. IT is the most important factor when the EDSS is below 7. Informal care is the most significant factor when the EDSS is above 7.5. IT accounts for 19% – 34% of total costs, depending on the percentage of patients treated (31% in our MS Unit, and 67.5% in the study sample).

Our results are consistent with other costs studies reviewed in the literature [19-32], which also showed that the costs of MS increased as disability progressed (Table 4). In our region, which has a public health care system, a significant part of the cost is covered by the patient's family, who act as unpaid caregivers. As in Italy[24], this is the culturally accepted mode of care for MS patients in Catalonia. Therefore, informal care expenses have been underestimated to date.

Despite the limitations described, cost-of-illness analyses like this study, which are based on real MS population data rather than on selected patients from trials, are important. They can be used to perform cost-effectiveness analyses which can demonstrate whether or not immunomodulatory agents are cost-saving in the long term, under real conditions of clinical practice.

## Conclusion

The present study reflects the significant social and healthcare impact of a chronic disease like MS. This impact increases with the progression of disability (EDSS).

The total mean societal costs of MS estimated in our sample, a cohort of MS patients monitored in our MS Unit in Baix Llobregat (Catalonia, Spain), were 24272 euros per patient/year. Costs ranged from 14327 euros (EDSS = 0) to 52841 euros (EDSS = 7.5–9.5). The direct costs were higher than indirect costs in all EDSS stages. This was mostly due to IT use in lower EDSS stages, and to informal care in higher EDSS stages.

Conclusions from this kind of analyses will be helpful for clinicians and for health care authorities, as cost-of-illness studies should be a first and indispensable step toward analysing the cost-effectiveness of IT. The cost-effectiveness of treatments is an increasingly important issue in policy decision-making which helps to optimise available resources.

## Competing interests

The author(s) declare that they have no competing interests.

## Authors' contributions

VC conceived of the study and participated in: its design, the data collection, the data analysis, and the draft of the manuscript. OC, LA, LR, EM, and LG participated in the data collection and contributed to the conception and design of the study. SMY and AMY participated in the design of the study and made substantial contributions to the data analysis and to the manuscript revision. TA coordinated the study, participated in its conception and its design, and reviewed the manuscript. All authors have read and approved the final manuscript.

## Pre-publication history

The pre-publication history for this paper can be accessed here:



## Figures and Tables

**Table 1:** The baseline characteristics of patients analysed.

	*N = 200*	*N = 254*	*p*
Mean age -years-	41.6	42.1	*0.6*
Sex (%female)	65.5	63.3	*0.6*
EDSS (mean)	2.7	2.8	*0.9*
EDSS (median)	2	2	*0.6*
Mean disease duration (years)	12.3	11.9	*0.7*
Clinical form of MS (% of RR/SP/PP)	79.5/13/7.5	81.9/8.7/9.4	*0.3*
Mean number of relapses during the previous year	0.3	0.3	*0.2*
% of patients on IT	67.5	45.7	*0.0*

No differences were found between patients who answered the questionnaire (N = 200) and patients who did not answer it (N = 254), except for the percentage of patients on IT.

MS: multiple sclerosis. RR: relapsing-remitting. SP: secondary progressive. PP: primary progressive. IT: immunomodulatory treatment

**Table 2:** The direct costs of MS (mean annual cost per patient per year, in *euros*).

*EDSS*	*0*	*1–3*	*3.5–5.5*	*6–7*	>*7.5*	
• Medical visits in hospital, due to MS	124	198	262.9	238.7	157.6	*204.4*
• *Medical visits in hospital, due to related diseases*	*10.4*	*33.7*	*01.1*	*78*	*03*	*43.2*
• Complementary tests in hospital, due to MS	10.3	160.6	29.1	436.4	8.5	*130.8*
• *Complementary tests in hospital, due to related diseases*	*14.5*	*10.0*	*10*	*05.2*	*33.0*	*24.5*
• Visits to General Practitioner, due to MS	0	93.5	173.2	26.7	206.7	*100.1*
• Hospitalization, due to MS	0	29.4	42.7	123	135.8	*42.6*
• Ambulance displacements, due to MS	0	21.9	0	330.5	170.3	*49.2*
• Taxi displacements, due to MS	0	52.5	196.8	27.5	70.9	*75.7*
• Home adaptations, due to MS	0	237.4	838.3	2071.3	7188	*874.3*
• Car adaptations, due to MS	0	56	285.7	705.9	1272.3	*220*
• Workplace adaptations, due to MS	0	50.2	35.2	240.6	269.1	*69.5*
• Rehabilitation, due to MS	0	368.2	1077.2	1201.8	796	*569.2*
• Pharmacological treatment, due to MS	20.6	39.8	81	44.6	66.2	*48.1*
• *Pharmacological treatment, due to related diseases*	*32.8*	*107.3*	*244.7*	*277.5*	*300.4*	*201.3*
• **Informal care**, due to MS	**1069**	**1856**	**5456**	**11555**	**22164**	***4831***
*informal care, when replacement costs vary*	*030–2122*	*1114–3713*	*3273–10011*	*0033–23111*	*13208–44328*	*2898–9661*
• **IMMUNOMODULATORY treatment**, due to MS	**6785**	**8815**	**9921**	**6584**	**4150**	***8377***
Total direct costs, due to MS	8009	11979	18399	23587	36656	*15591*
*Total directcosts, due to related diseases*	*07*	*242*	*325*	*451*	*400*	*269*
**TOTAL DIRECT COSTS**	**8706**	**12221**	**18724**	**24037**	**37062**	***15860***
*Total directcosts IT Informal care value = 30% MHW*	*8273*	*11479*	*16541*	*19415*	*28196*	*13927*
*Total directcosts IT Informal care value = 100% MHW*	*9759*	*14078*	*24179*	*35593*	*59226*	*20690*
% of overall costs of MS attributable to direct costs	61	65	66	58	70	*65*

Each column reflects the data obtained from 5 different groups of patients, classified according to thier EDSS stage. The last column shows data calculated from the whole sample. A sensitivity analysis was performed, by varying the replacement costs used to value informal care (taken as either 30% or 100% of the minimun hourly wage [*MHW*])

**Table 3:** The indirect costs of MS (the mean annual cost per patient per year, in *euros*).

*EDSS*	*0*	*1.5–3.0*	*3.5–5.5*	*6.0–7.0*	*>7.5*	
Productivity losses in patients, due to:						
**1) Short term sickness absences**, because of MS/others	902/0	1415/1155	1683/358	2113/0	0/0	*1394/693*
*Sick leaves (number of days/year) because of MS*	*6.04*	*13.48*	*16.3*	*21.47*	*0*	*13.15*
*Sick leaves (number of days/year) because of other reasons*	*0.48*	*12.9*	*4.33*	*0*	*0*	*7.9*
*Work absences (number of hours/year) because of MS*	*35.04*	*14.65*	*30.2*	*0*	*0*	*18.2*
						
**2) Long term sickness absences **(change in work-status)	4719	4046	7555	15048	15779	*6326*
*% p. with premature work invalidity due to MS*	*0*	*15*	*38*	*70*	*82*	*26.5*
*% p. with premature retirement due to MS*	*0*	*0.9*	*0*	*0*	*9*	*1*
*% p. with reduction of worktime hours due to MS*	*4*	*1.8*	*2.4*	*0*	*0*	*2*
*% p. who changes-adaptates their work-status to their MS*	*13*	*4.7*	*4.7*	*0*	*0*	*5*
*% p. who do not change their work-status, despite their- MS*	*4*	*9*	*4.7*	*6*	*0*	*7*
						
**TOTAL indirect costs due strictily to MS**	**5621**	**5461**	**9238**	**17161**	**15779**	***7719***
						
**TOTAL INDIRECT costs **(due to MS and related reasons)	**5621**	**6616**	**9596**	**17161**	**15779**	***8412***
**% of total costs of MS **attributable to **indirect costs**	39	35	34	42	30	***35***

Each column represents the costs calculated for each disability group of patients (*p*.), established according to their EDSS stages. The last column shows the costs calculated for the whole sample (N = 200). The number of work absences and the percentage of patients who changed their work-status are also detailed, according to EDSS stages.

**Table 4:** The total costs of MS, in euros per patient per year, for the sample studied (N = 200) according to disability.

*EDSS*	*0*	*1.0–3.0*	*3.5–5.5*	*6.0–7.0*	*7.5–9.5*	*mean*
**total costs (direct + indirect)**	**14327**	**18837**	**27869**	**41198**	**52841**	***24272***
						
% patients on IM treatment our MS Unit	6	57	41	47	4	*31*
**total costs adjusted by % of patients on IT in our MS Unit**	**7400**	**17589**	**23157**	**40273**	**48729**	***19589***
% of global costs attributable to IT, in MS Unit	9	43	21	14	1	***19***
						
*Disability -according to EDSS*-	*mild*		*moderate*		*severe*	
Global costs of MS in Poland (Orlewska et al., 2005)	6572		9362		11078	
"Italy (Amato et al, 2002)	7463		23088		35975	
"Sweden (Heriksson et al., 2001)	18787		36470		91986	
"Germany (Kobelt et al., 2000)	20000		39000		61000	
"United Kingdom (Kobelt et al., 2000)	20000		38000		41500	
"France (Murphy et al., 1998)	8000		16000		22400	
"Germany (Murphy et al., 1998)	9088		8224		22804	
"United Kingdom (Murphy et al., 1998)	20500		27004		58488	

Costs were calculated for patients classified in the 5 EDSS groups (columns 1 to 5) and for the whole sample, N = 200, (column 6). The total costs were adjusted by the percentage of patients on IT (immunomodulatory treatment) in our MS Unit in 2004. Costs that have been reported in other countries have been converted into euros and are described below (also according to patients' disability).
